# VEGFR-1 Regulates EGF-R to Promote Proliferation in Colon Cancer Cells

**DOI:** 10.3390/ijms20225608

**Published:** 2019-11-09

**Authors:** Hikaru Nagano, Chisato Tomida, Naoko Yamagishi, Shigetada Teshima-Kondo

**Affiliations:** 1 Department of Medical Nutrition, Osaka Prefecture University, 3-7-30 Habikino, Habikino, Osaka 583-8555, Japan; 2 Department of Food and Nutrition, Faculty of Home Economics, Tokyo Kasei University, 1-18-1 Kaga, Itabashi, Tokyo 173-8602, Japan; 3 Department of Anatomy and Cell Biology, School of Medicine, Wakayama Medical University, 811-1 Kimiidera, Wakayama 641-8509, Japan

**Keywords:** vascular endothelial growth factor receptor-1 (VEGFR-1), vascular endothelial growth factor (VEGF), epidermal growth factor receptor (EGF-R), proliferation, colon cancer cells

## Abstract

The relationship between epidermal growth factor (EGF) and vascular endothelial growth factor (VEGF) pathways in tumor growth is well established. EGF induces VEGF production in cancer cells, and the paracrine VEGF activates vascular endothelial cells to promote tumor angiogenesis and thus supports tumor cell growth in an angiogenesis-dependent manner. In this study, we found angiogenesis-independent novel crosstalk between the VEGF and the EGF pathways in the regulation of colon cancer cell proliferation. Stimulation of colon cancer cells with VEGF-A and placental growth factor (PlGF) activated VEGF receptor-1 (VEGFR-1) and increased proliferation activity in an autocrine EGF/EGF receptor (EGF-R)-dependent manner. Mechanistically, VEGFR-1 interacted with and stabilized EGF-R, leading to increased EGF-R protein levels and prolonged its expression on cell surface plasma membrane. In contrast, VEGFR-1 blockade by a neutralizing antibody and an antagonistic peptide of VEGFR-1 suppressed the complex formation of VEGFR-1 and EGF-R and decreased EGF-R expression via a lysosome-dependent pathway, resulting in the suppression of proliferation activity. Our results indicated that VEGFR-1 regulated EGF-R expression to promote proliferation activity in a cell-autonomous-dependent manner.

## 1. Introduction

Tumor angiogenesis, the growth of new capillary blood vessels, is a hallmark of cancer and is essential for tumor growth and progression [1,2,3]. Vascular endothelial growth factor (VEGF) is a critical factor for tumor angiogenesis in numerous solid malignancies, and tumor cells overexpress and secrete VEGF. Paracrine VEGF acts on vascular endothelial cells and induces their proliferation, differentiation and migration, resulting in angiogenesis and providing oxygen and nutrients to the tumor [2,3]. The importance of VEGF-induced angiogenesis in tumor growth is strongly supported by studies showing that blockade of VEGF and its receptors results in decreased angiogenesis and subsequent abrogation of cancer growth [2,3,4].

The VEGF family of ligands includes VEGF-A, -B, -C, -D and placental growth factor (PlGF) [5,6]. PlGF and VEGF-B interact with vascular endothelial growth factor receptor-1 (VEGFR-1), whereas VEGF-A is able to bind to VEGFR-1 and VEGFR-2 [5,6]. In endothelial cells, VEGFR-2 is known to translate the full range of VEGF-A responses, i.e., regulating endothelial survival, proliferation, migration and formation of the vascular tube [5,6]. In contrast, VEGFR-1 binds VEGF-A with higher affinity than dose VEGFR-2, however, VEGFR-1 has a 10-fold lower kinase activity than VEGFR-2 in endothelial cells. Therefore, VEGFR-1 is known to function as a negative regulator of VEGF-A signaling in endothelial cells [5,6].

VEGFR-1 was initially believed to be expressed by only endothelial cells; however, recent studies, including from us, have demonstrated that VEGFR-1 is also expressed on a variety of tumor cells, including colon, breast, gastric, osteosarcoma, pancreatic, melanoma, prostate and ovarian cancers [7,8,9,10,11,12,13,14,15,16,17,18]. Although the biological function of VEGFR-1 on cancer cells is not fully understood, the concomitant expression of VEGF ligands and VEGFR-1 by tumor cells suggests that an autocrine VEGF/VEGFR-1 signaling loop exists [7,8,9]. This autocrine pathway is thus considered to be an angiogenesis-independent function of VEGF. In fact, VEGF/VEGFR-1 autocrine signaling regulates the proliferation and growth of several types of cancer cells, including breast, osteosarcoma, melanoma, ovarian and skin cancers [11,13,15,16,17]. For example, VEGF-A and PlGF stimulated cell proliferation through VEGFR-1 in breast cancer cells [11]. In osteosarcoma cells, autocrine VEGF-A signaling induced constitutive activation of VEGFR-1, resulting in increased proliferation and survival activities [13]. The angiogenesis-independent cell proliferation effect in vivo has been well demonstrated in a K5-SOS conditional knockout mouse model [17]. When VEGFR-1 was genetically depleted in epidermal cancer cells but not in vascular endothelial cells in the mice, cancer cell proliferation was decreased, resulting in the suppression of skin tumor development [17].

However, the precise mechanism by which VEGFR-1 elicits cell proliferation is not yet fully understood. Here we show that VEGFR-1 interacted with and stabilized epidermal growth factor receptor (EGF-R), leading to an increased EGF-R-dependent proliferative activity of colon cancer cells. Thus, we revealed novel crosstalk between the VEGF and the EGF pathways in colon cancer cells.

## 2. Results

### 2.1. Effect of VEGFR-1 Activation on Cell Proliferation in Colon Cancer Cells

**Figure 1:**
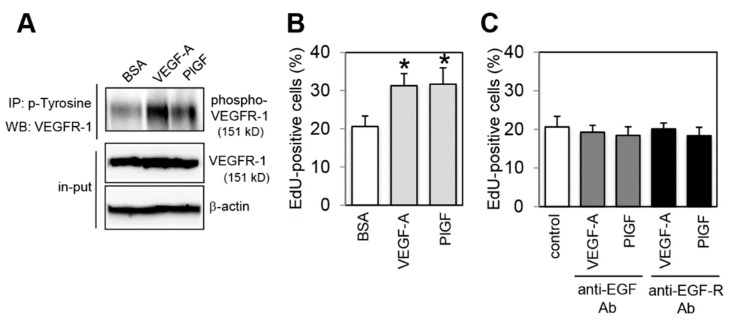
Vascular endothelial growth factor receptor-1 (VEGFR-1) activation results in increased proliferation activity that depends on autocrine epidermal growth factor (EGF)/EGF receptor (EGF-R) pathway. (**A**) Activation of VEGFR-1 by vascular endothelial growth factor-A (VEGF-A) and placental growth factor (PlGF) stimulation. Cells were treated with VEGF-A, (PlGF) or control bovine serum albumin (BSA) for 5 min. Phosphorylated VEGFR-1 was detected by immunoprecipitation with an anti-phospho-tyrosine antibody and immunoblotting with an anti-VEGFR-1 antibody. The same lysates (10% input) were immunoblotted with an anti-VEGFR-1 antibody to normalize the amounts of each sample. The levels of β-actin are shown as a loading control. (**B**) Quantification of EdU (5-ethynyl-2’-deoxyuridine) positive cells under VEGFR-1 activating conditions. Data are indicated by means ± SD (*n* = 6–8). * p < 0.01, statistically significant increase compared with the BSA-treated control cells. (**C**) Quantification of EdU positive cells under EGF/EGF-R inhibiting conditions. Cells were pretreated with neutralizing antibodies against EGF (anti-EGF Ab) and EGF-R (anti-EGF-R Ab), or control non-immune IgG (control) for 1 h, and then treated with VEGF-A or PlGF for 24 h. Data are indicated by means ± SD (*n* = 6–8).

There are several reports showing that human colon cancer HCT116 cells express VEGFR-1 [10,12,18]. Here, we confirmed that the VEGFR-1 was expressed on cell surface and functional in HCT116 cells. Flow cytometry analysis showed that VEGFR-1 was expressed on cell surface of HCT116 cells (Figure S1A). Stimulation of cells with VEGF-A and PlGF increased VEGFR-1 phosphorylation levels (Figure 1A). We also examined the expression of VEGFR-2 and found that HCT116 cells weakly expressed VEGFR-2 (Figure S1A,B).

We then examined the effect of VEGFR-1 activation on the proliferation activity of HCT116 cells using a modified thymidine analogue EdU (5-ethynyl-2’-deoxyuridine) incorporation assay. The result shown in Figure 1B clearly indicated that VEGF-A and PlGF treatment significantly increased the number of EdU-positive proliferating cells compared with bovine serum albumin (BSA) control treatment. We also examined whether VEGFR-2 was involved in the VEGF-A-stimulated proliferation activity using a VEGFR-2 specific inhibitor (ZM323881) [19]. Treatment of cells with ZM323881 did not affect both basal and VEGF-A-stimulated proliferation (Figure S1C). These results indicate that VEGF-A-induced proliferation was mediated by VEGFR-1, but not by VEGFR-2.

In colon cancer cells, autocrine EGF signaling is a well-known critical pathway that activates proliferation. In addition, it has been reported that crosstalk between EGF and VEGF-A signaling exists in tumor growth [20,21,22]. Thus, we hypothesized that an autocrine EGF/EGF-R pathway may be involved in the VEGFR-1 induced increase in cell proliferation activity. To address this hypothesis, autocrine EGF-R loop was blocked using neutralizing antibodies against EGF ligand (anti-EGF Ab) and against EGF-R (anti-EGF-R Ab) under VEGFR-1 activating conditions. Inhibition of EGF or EGF-R completely attenuated the proliferation activity induced by VEGF-A and PlGF stimulation (Figure 1C). These results indicated that an increase in proliferation activity induced by VEGFR-1 activation was mediated by autocrine EGF/EGF-R pathway.

### 2.2. Effect of VEGFR-1 Activation on EGF-R Expression

**Figure 2:**
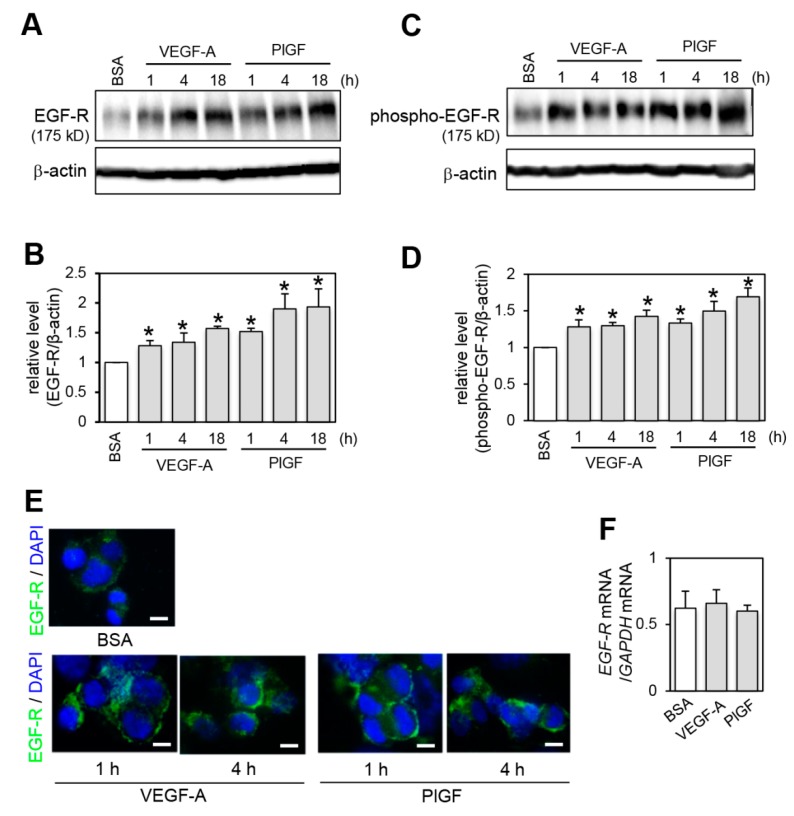
VEGFR-1 activation results in increased EGF-R expression levels. (**A**–**D**) Cells were treated with control BSA for 18 h, or with VEGF-A or PlGF for the indicated times. EGF-R (**A**) and phosphorylated EGF-R (**C**) levels were determined by immunoblot analysis. The levels of β-actin are shown as a loading control. Quantification of EGF-R levels (**B**) and phosphorylated EGF-R levels (**D**) normalized to β-actin from three independent experiments. * *p* < 0.01, statistically significant increase compared with the BSA-treated control. (**E**) Immunofluorescent staining with cell surface EGF-R. Cells were pre-treated with control BSA for 4 h or with VEGF-A and PlGF for the indicated times. Living cells were then incubated with an anti-EGF-R antibody conjugated with FITC for 30 min at 4 degrees and fixed. Nuclei were stained with 4′,6-diamidino-2-phenylindole (DAPI). Representative fluorescent images are shown. Scale bar = 10 μm. (**F**) Expression levels of *EGF-R* mRNA were determined by RT-qPCR analysis. Values were normalized for the amount of *GAPDH* mRNA (*n* = 5, means ± SD).

As recent studies demonstrated that several growth factors, such as HGF and PDGF, regulate EGF-R expression at the protein level and affect cell proliferation [23,24,25], we investigated whether VEGF-A and PlGF affected EGF-R protein expression levels by immunoblot analysis. EGF-R levels were rapidly up-regulated by VEGF-A and PlGF stimulation within 1 h, and the increase continued in a time-dependent manner compared with the BSA control treatment (Figure 2A,B). We further examined whether VEGFR-1 actually up-regulated EGF-R activation (phosphorylation) by immunoblot analysis with an anti-phospho-EGF-R antibody. In correlation with the elevation of EGF-R protein levels, VEGF-A and PlGF stimulation increased and prolonged EGF-R phosphorylated levels (Figure 2C,D).

To examine whether the increased EGF-R was expressed on cell surface plasma membrane to receive a continuous extracellular EGF proliferation signal, we performed immunofluorescence staining using an anti-EGF-R antibody recognizing the extracellular domain of the receptor. In agreement with the immunoblotting result (Figure 2A), treatment with VEGF-A and PlGF significantly prolonged *EGF-R* expression on the cell surface compared to control BSA treatment (Figure 2E). We determined the effect of VEGFR-1 activation on *EGF-R* mRNA expression levels by RT-qPCR analysis and found that the levels were not significantly changed by VEGF-A and PlGF stimulation (Figure 2F). These observations suggest that VEGFR-1 activation increased EGF-R protein stability.

### 2.3. Effect of VEGFR-1 Activation on EGF-R Stability

**Figure 3:**
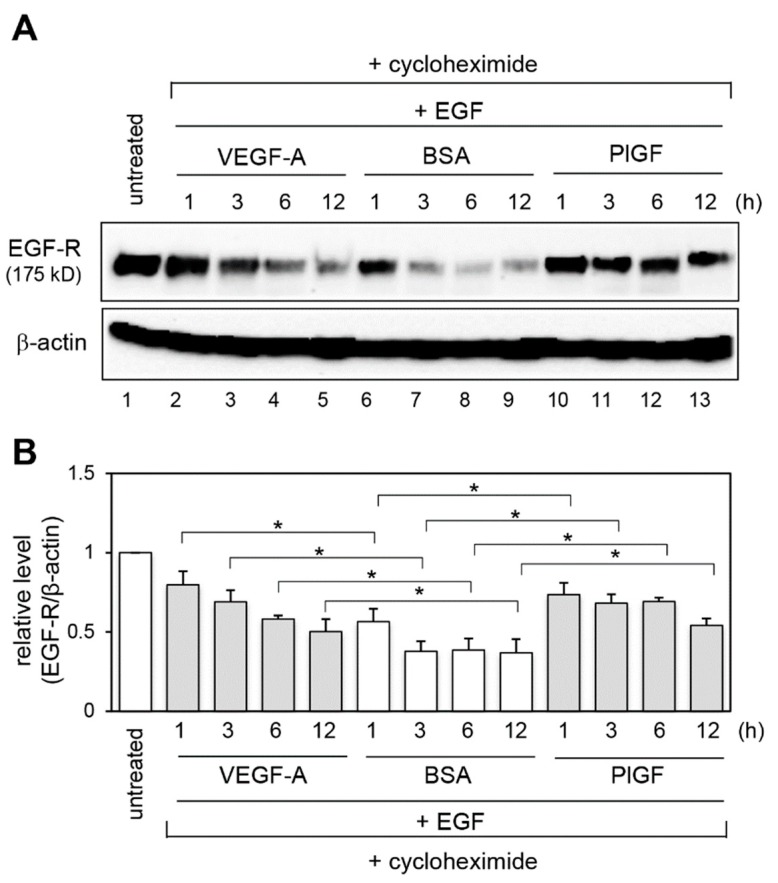
VEGFR-1 activation stabilizes EGF-R. (**A**) Cells were pretreated with control BSA, VEGF-A or PlGF for 1 h, then incubated with EGF plus cycloheximide for the indicated times. EGF-R protein levels were determined by immunoblot analysis. The levels of β-actin are shown as a loading control. (**B**) Quantification of EGF-R levels normalized to β-actin from three independent experiments. * *p* < 0.01, statistically significant increase compared with the BSA-treated control cells at the corresponding each time point.

To address whether the stability of EGF-R protein was increased by VEGFR-1 activation, we performed cycloheximide (a protein synthesis inhibitor) chase assay using an EGF-stimulated EGF-R degradation model [26,27]. Cells were pretreated with VEGF-A, PlGF or BSA for 1 h, then treated with EGF and cycloheximide for the indicated times. The cycloheximide chase assay indicated that the BSA-treated cells showed a time-dependent decrease in EGF-R expression levels upon EGF stimulation (Figure 3A, lanes 6–9, and Figure 3B). In contrast, VEGF-A and PlGF stimulation clearly increased EGF-R stabilization (Figure 3A, lanes 2–5 and 10–13, and Figure 3B).

### 2.4. Effect of VEGFR-1 Activation on Interaction of VEGFR-1 and EGF-R

**Figure 4:**
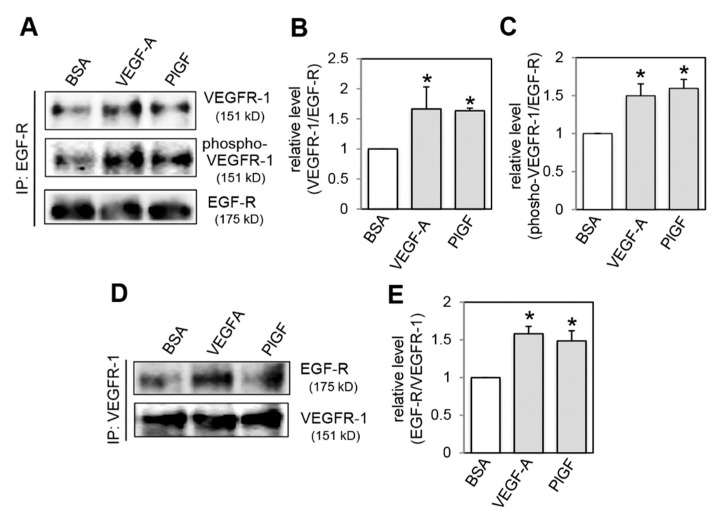
VEGFR-1 activation increases complex formation of VEGFR-1 and EGF-R. (**A**) Cells were treated with BSA, VEGF-A or PlGF for 30 min. Cell lysates were immunoprecipitated with an anti-EGF-R antibody (IP: EGF-R) and then immunoblotted for VEGFR-1 (**A**, upper panel) and for phosphorylated VEGFR-1 (**A**, middle panel). In parallel, Western blot was performed to control for EGF-R concentration in the immunoprecipitates (**A**, lower panel). (**B**,**C**) Quantification of co-immunoprecipitated VEGFR-1 levels (**B**) and phosphorylated VEGFR-1 levels (**C**) normalized to immunoprecipitated EGF-R levels from three independent experiments. * *p* < 0.01, statistically significant increase compared with the BSA-treated control. (**D**) Cells were treated with BSA, VEGF-A or PlGF for 30 min. Cell lysates were immunoprecipitated with an anti-VEGFR-1 antibody (IP: VEGFR-1) and then immunoblotted for EGF-R (**D**, upper panel). In parallel, Western blot was performed to control for VEGFR-1 concentration in the immunoprecipitates (**D**, lower panel). (**E**) Quantification of co-immunoprecipitated EGF-R levels normalized to immunoprecipitated VEGFR-1 levels from three independent experiments. * *p* < 0.01, statistically significant increase compared with the BSA-treated control.

A recent report demonstrated that PDGF-Rβ prolongs EGF-R expression on cell surface by the formation of a PDGF-R β/EGF-R heterodimer [25]. Thus, we hypothesized that EGF-R stabilization on cell surface may be induced by the interaction of VEGFR-1 with EGF-R. To test this hypothesis, immunoprecipitation in combination with immunoblotting analysis was performed (Figure 4). Immunoprecipitation results with an anti-EGF-R antibody showed that the basal interaction of VEGFR-1 with EGF-R was observed in control BSA treated cells (Figure 4A). Treatment with VEGF-A and PlGF increased their interaction (Figure 4A,B). Immunoblot analysis with an anti-phosphorylated VEGFR-1 antibody showed that phosphorylated VEGFR-1 interacted with EGF-R (Figure 4A,C). Reciprocal immunoprecipitation with an anti-VEGFR-1 antibody confirmed that VEGFR-1 activation induced the increase in VEGFR-1 and EGF-R complex formation (Figure 4D,E).

### 2.5. Effect of VEGFR-1 Blockade on Cell Proliferation Activity

**Figure 5:**
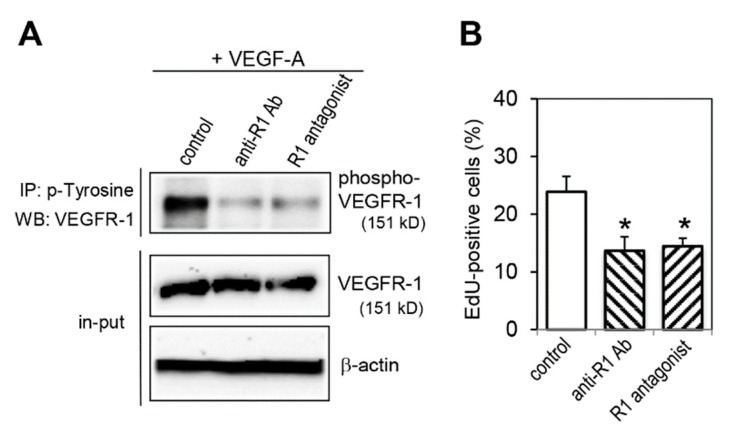
VEGFR-1 blockade decreases proliferation activity. (**A**) Inhibition of VEGFR-1 by anti-VEGFR-1 neutralizing antibody (anti-R1 Ab) and VEGFR-1 antagonist (R1 antagonist). Cells were treated with anti-R1 Ab, R1 antagonist or control IgG (control) in the presence of VEGF-A for 5 min. Phosphorylated VEGFR-1 was detected as described in legend to Figure 1. The same lysates (10% input) were immunoblotted with an anti-VEGFR-1 antibody to normalize the amounts of each sample. The levels of β-actin are shown as a loading control. (**B**) Quantification of EdU positive cells under VEGFR-1 inhibiting conditions. Cells were treated with anti-R1 Ab, R1 antagonist or control IgG for 24 h. Data are indicated by means ± SD (*n* = 6–8). * p < 0.01, statistically significant decrease compared with the control cells.

We then examined the effect of VEGFR-1 inhibition on cell proliferation activity using two different VEGFR-1 inhibitors, a neutralizing anti-VEGFR-1 antibody (anti-R1 Ab) and an antagonistic peptide for VEGFR-1 (R1 antagonist) [28]. We confirmed that treatment with anti-R1 Ab and R1 antagonist significantly reduced VEGFR-1 phosphorylation upon VEGF-A stimulation (Figure 5A). Compared with control IgG treatment, treatment with anti-R1 Ab and R1 antagonist significantly decreased proliferation activity (Figure 5B).

### 2.6. Effect of VEGFR-1 Blockade on EGF-R Expression

**Figure 6:**
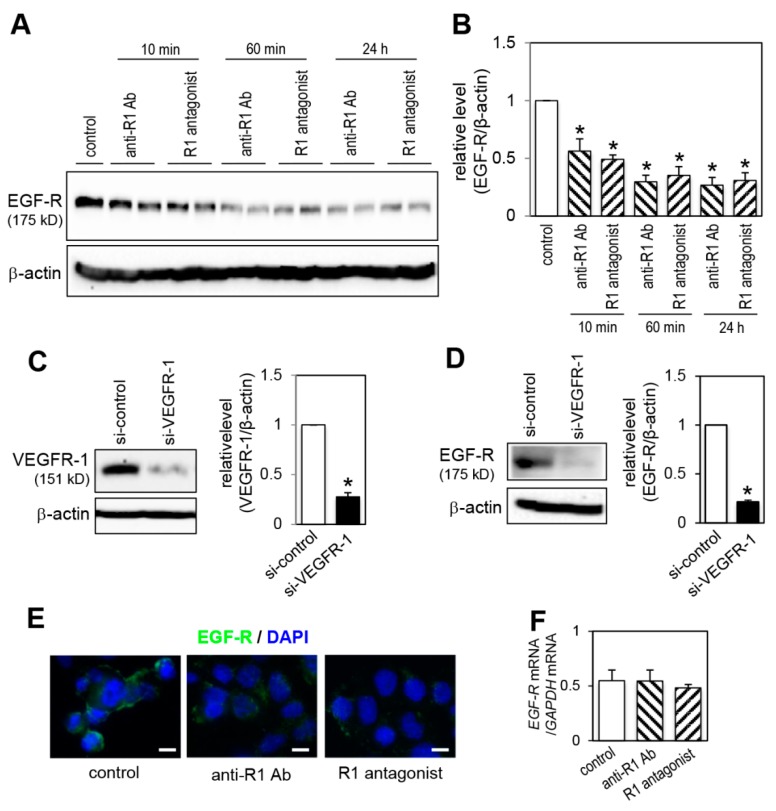
VEGFR-1 blockade decreases EGF-R expression. (**A**) Cells were treated with anti-R1 Ab and R1 antagonist for the indicated times, or with a control IgG for 24 h (indicated by control). EGF-R protein levels were determined by immunoblot analysis. The levels of β-actin are shown as a loading control. (**B**) Quantification of EGF-R levels normalized to β-actin from three independent experiments. * *p* < 0.01, statistically significant decrease compared with the control cells. (**C**,**D**) Cells were transfected with the indicated siRNA for 24 h. Levels of VEGFR-1 protein (**C**, left) and EGF-R protein (**D**, left) were determined by immunoblot analysis. Quantification of VEGFR-1 levels (**C**, right) and EGF-R levels (**D**, right) normalized to β-actin from three independent experiments. * p < 0.01, statistically significant decrease compared with the si-control-transfected cells. (**E**) Immunofluorescent staining with cell surface EGF-R. After cells were treated with anti-R1 Ab, R1 antagonist or control IgG for 1 h, cell surface EGF-R was stained as described in legend to Figure 2E. Scale bar = 10 μm. (**F**) Cells were treated with anti-R1 Ab, R1 antagonist or control IgG for 12 h. Expression levels of *EGF-R* mRNA were determined by RT-qPCR analysis. Values were normalized for the amount of *GAPDH* mRNA (*n* = 5, means ± SD).

We then examined the effect of VEGFR-1 inhibition on EGF-R expression levels. Treatment of cells with anti-R1 Ab and R1 antagonist rapidly reduced the EGF-R levels within 10 min (Figure 6A,B). The EGF-R levels were further decreased after 60 min of VEGFR-1 blockade, and the decrease continued to 24 h (Figure 6A,B). Knockdown of VEGFR-1 using a siRNA targeting *VEGFR-1* mRNA for 24 h (Figure 6C) also significantly reduced EGF-R expression (Figure 6D). We further examined EGF-R expression on the plasma membrane by immunofluorescence staining. In agreement with immunoblotting result (Figure 6A), blockade of VEGFR-1 decreased the cell surface EGF-R expression compared to the control IgG-treated conditions (Figure 6E). We determined the effect of VEGFR-1 inhibition on *EGF-R* mRNA expression levels by RT-qPCR analysis and found that the levels were not significantly changed by VEGFR-1 blockade (Figure 6F).

### 2.7. Effect of EGF-R Knockdown on Proliferation Activity

**Figure 7:**
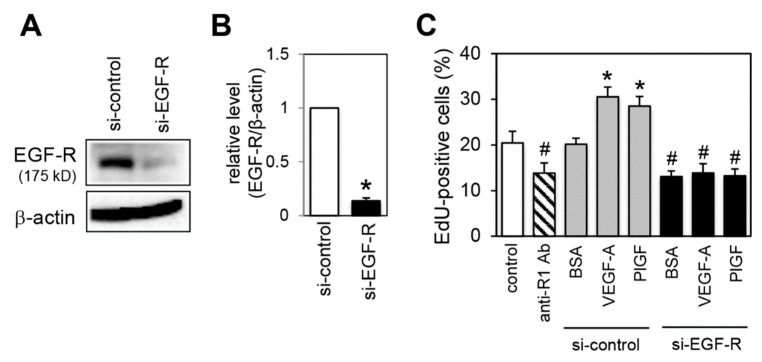
EGF-R knockdown blocks VEGFR-1 activation-induced proliferation activity. (**A**) Cells were transfected with the indicated siRNA for 24 h. EGF-R protein levels were determined by immunoblot analysis. The levels of β-actin are shown as a loading control. (**B**) Quantification of EGF-R levels normalized to β-actin from three independent experiments. * *p* < 0.01, statistically significant decrease compared with the si-control-treated cells. (**C**) Quantification of EdU positive cells under EGF-R silencing conditions. Cells were transfected with the indicated siRNA for 24 h, then treated with VEGF-A, PlGF or BSA for the additional 24 h. For a control experiment, cells were treated with only anti-R1 Ab or control IgG (indicated by control) for 24 h. Data are indicated by means ± SD (*n* = 6–8). ^#^ *p* < 0.01, statistically significant decrease compared with the control. * *p* < 0.01, statistically significant increase compared with the control.

To confirm that EGF-R was essential for VEGFR-1-mediated proliferation activity, we examined the effect of EGF-R silencing on the proliferation. Transfection of cells with a siRNA targeting *EGF-R* mRNA significantly reduced EGF-R protein expression compared with control siRNA transfected cells (Figure 7A,B). Knockdown of EGF-R decreased basal proliferation activity to the same extent as VEGFR-1 blockade (Figure 7C). Furthermore, EGF-R silencing completely attenuated the VEGF-A- and PlGF-stimulated proliferation (Figure 7C).

### 2.8. Effect of a Lysosomal and Proteasomal Inhibitor on EGF-R Expression

**Figure 8:**
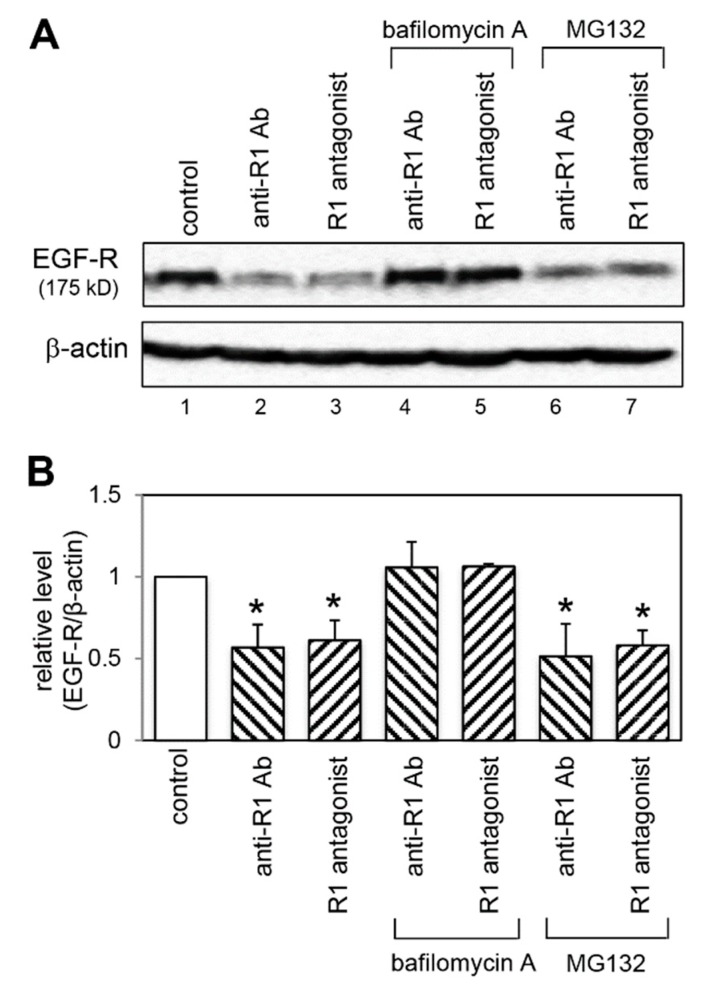
EGF-R downregulation is mediated by a lysosomal degradation pathway under VEGFR-1 inhibited conditions. (**A**) Cells were pretreated with a lysosomal inhibitor (bafilomycin A) or a proteasomal inhibitor (MG132) for 1 h, then treated with anti-R1 Ab (lanes 4 and 6) and R1 antagonist (lanes 5 and 7) for 1 h. Without bafilomycin A or MG132, cells were treated with anti-R1 Ab alone (lane 2), R1 antagonist alone (lane 3) or control IgG alone (lane 1, indicated by control) for 1 h. EGF-R protein levels were determined by immunoblot analysis. The levels of β-actin are shown as a loading control. (**B**) Quantification of EGF-R levels normalized to β-actin from three independent experiments. * *p* < 0.01, statistically significant decrease compared with the control.

The rapid decrease in EGF-R protein levels observed in Figure 6A suggested that VEGFR-1 inhibition led to EGF-R degradation. Downregulation of EGF-R protein has been shown to occur predominantly through lysosomal and/or proteasomal degradation pathways [26,27]. To determine which degradation pathway was involved in the EGF-R downregulation under VEGFR-1 blocking conditions, we used bafilomycin A (a lysosome inhibitor) and MG132 (a proteasome inhibitor). The result indicated in Figure 8A,B showed that the EGF-R reduction was completely inhibited by bafilomycin A (lanes 4 and 5), but not by MG132 (lanes 6 and 7). These results indicated that the EGF-R downregulation was mediated by a lysosomal degradation pathway.

### 2.9. Effect of VEGFR-1 Blockade on Interaction of VEGFR-1 and EGF-R

**Figure 9:**
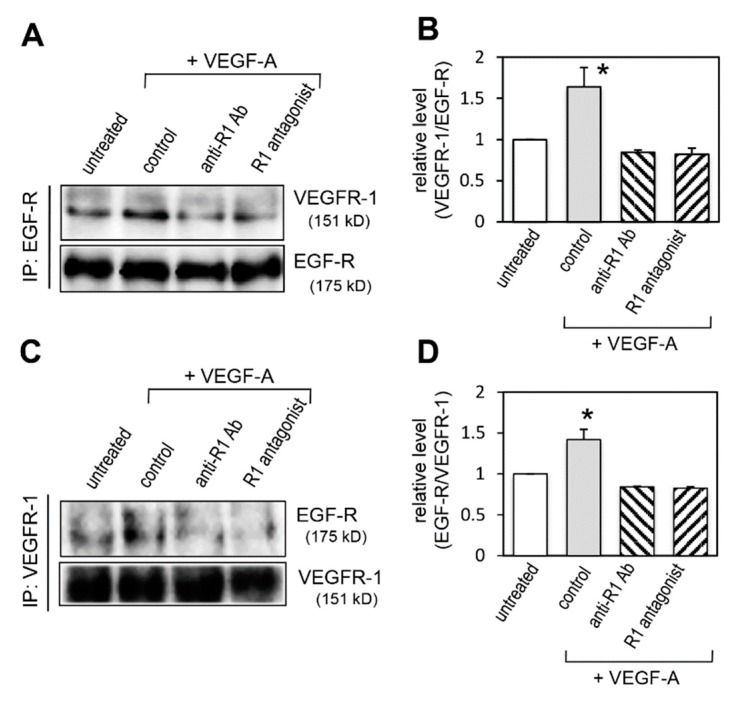
VEGFR-1 blockade decreases complex formation of VEGFR-1 and EGF-R. Cells were untreated or pretreated with anti-R1 Ab, R1 antagonist or control IgG (control) for 3 min, and then stimulated with VEGF-A for 2 min. (**A**) Cell lysates were immunoprecipitated with an anti-EGF-R antibody (IP: EGF-R) and then immunoblotted for VEGFR-1 (**A**, upper panel). In parallel, Western blot was performed to control for EGF-R concentration in the immunoprecipitates (**A**, lower panel). (**B**) Quantification of co-immunoprecipitated VEGFR-1 levels normalized to immunoprecipitated EGF-R from three independent experiments. * *p* < 0.01, statistically significant increase compared with the untreated cells. (**C**) Cell lysates were immunoprecipitated with an anti-VEGFR-1 antibody (IP: VEGFR-1) and then immunoblotted for EGF-R (**C**, upper panel). In parallel, Western blot was performed to control for VEGFR-1 concentration in the immunoprecipitates (**C**, lower panel). (**D**) Quantification of co-immunoprecipitated EGF-R levels normalized to immunoprecipitated VEGFR-1 from three independent experiments. * *p* < 0.01, statistically significant increase compared with the untreated cells.

To examine whether VEGFR-1 inhibition decreased the interaction of VEGFR-1 and EGF-R, immunoprecipitation in combination with immunoblotting was performed. Immunoprecipitation with anti-EGF-R antibody showed that the increased interaction of VEGFR-1 with EGF-R upon VEGF-A stimulation was decreased by VEGFR-1 blockers (Figure 9A,B). Reciprocal immunoprecipitation with anti-VEGFR-1 antibody confirmed the decrease in their interaction (Figure 9C,D). These results suggest that EGF-R destabilization induced by VEGFR-1 blockade was mediated by the dissociation of the VEGFR-1 and EGF-R complex.

## 3. Discussion

It has been demonstrated that there is angiogenesis-dependent crosstalk between the EGF and the VEGF pathway in several types of cancer cells [20]. Autocrine EGF signaling consistently induces VEGF-A expression and secretion by colon, gastric and breast cancer cells, leading to a paracrine VEGF-A-induced tumor angiogenesis that supports tumor growth and progression [21,22,29]. In contrast, inhibition of EGF signaling decreases VEGF-A production and thus inhibits angiogenesis and tumor growth [20,21,29].

In the present study, we elucidated novel angiogenesis-independent crosstalk between the VEGF and the EGF pathways in colon cancer cells. Activation of VEGFR-1 by VEGF-A and PlGF stabilized EGF-R protein and prolonged its expression and phosphorylation, resulting in increased autocrine EGF/EGF-R-dependent proliferation activity. In contrast, blockade of VEGFR-1 led to EGF-R destabilization, resulting in the suppression of proliferation activity. Thus, our study suggests that VEGFR-1 regulates cancer cell growth via two different pathways: an angiogenesis-independent autocrine pathway and an angiogenesis-dependent paracrine pathway.

In endothelial cells, VEGFR-1 has been reported to be implicated as a negative regulator of VEGFR-2 signaling for proliferation [5,6]. In contrast, in several types of cancer cells, VEGFR-1 promotes proliferation signal [9,10,11,12,13,14,15,16]. These findings suggest that involvement of VEGFR-1 in mitogenic signal is distinctly different between endothelial cells and cancer cells.

In cancer cells, it has been speculated that VEGFR-1 signaling relies on abnormal link between unrelated receptors that function synergistically to enhance proliferation [30]. For example, disruption of VEGF-A and VEGFR-1 genes in epidermal tumor cells decreases their proliferation [17]. Furthermore, VEGF-A disruption in an EGF-R-deficient background completely inhibits epidermal tumor growth, suggesting that there is an abnormal contribution of the VEGF-A and the EGF pathways in the tumor cells [17]. In this study, we provide the first demonstration that a direct interaction of VEGFR-1 and EGF-R contributed colon cancer cell proliferation.

It is well established that the control of EGF-R trafficking and degradation is regulated mainly by its ligands [27,28]. Binding of EGF to EGF-R leads to internalization of EGF-R and subsequent degradation by lysosome and/or proteasome pathways [27,28]. In addition to EGF, it has recently been reported that HGF controls EGF-R degradation [23,24]. The HGF/cMet system accelerates EGF-R degradation by inhibiting the binding of SHIP2 (Src homology domain 2-containing inositol 5′-phosphatase 2) to EGF-R, which is required to block the EGF-R degradation pathway [23].

In this study, we demonstrated novel growth factors that regulate EGF-R stabilization. The VEGF-A and PlGF/VEGFR-1 system stabilized EGF-R via the interaction of VEGFR-1 with EGF-R. In contrast, blockade of VEGFR-1 decreased their interaction and led to destabilization of EGF-R through a lysosome-dependent pathway. In addition to VEGFR-1, a recent report demonstrated that PDGF-Rβ formed a heterodimer with EGF-R and stabilizes EGF-R on the cell surface [25]. Thus, the stabilization of EGF-R is regulated by several growth factors and their receptors.

Recently, the concept of molecular targeted therapy for the treatment of colorectal cancer has emerged. Two critical molecular targets for the treatment of colorectal cancer are EGF-R and VEGF-A, because these two molecules are often overexpressed and are associated with inferior outcomes [4,31]. Thus, anti-EGF-R monoclonal antibodies (cetuximab and panitumumab) and an anti-VEGF-A monoclonal antibody (bevacizumab) are mainly used as therapies for colorectal cancer patients [31]. Despite the initial clinical efficacy of the anti-EGF-R agents against colorectal cancer, acquired resistance develops during therapy [31]. The resistance is caused by the overexpression of VEGF-A, PlGF and VEGFR-1 and thus the activation of alternative VEGFR-1 signaling pathway [32]. In the case of anti-VEGF-A therapy, acquired resistance is also developed via alternative PlGF/VEGFR-1 pathway activation [9,33,34]. The therapeutic evidence suggests that VEGFR-1 targeting therapy provides a promising strategy for reducing the risk of the acquired resistance.

In fact, VEGFR-1 targeted therapy has been recently found to block cancer growth by reducing the effects of VEGF-A and PlGF on cancer cells [9]. In addition to the direct anticancer effect, the VEGFR-1 blocking agents inhibit tumor angiogenesis by blocking the effects of VEGF-A and PlGF on vascular endothelial cells [9]. Intriguingly, in clinical studies, VEGFR-1 targeting therapies have been shown to be of potential benefit in patients with colorectal cancer [35,36,37,38,39]. For example, a VEGFR-1 blocking agent (aflibercept) that traps VEGF-A and PlGF [39,40] improved overall survival compared with a placebo in patients with metastatic colorectal cancer in a clinical study [36,37,38,39]. This benefit may be due to the inhibition of both colorectal cancer cells and vascular endothelial cells. Thus, the molecular elucidation of VEGFR-1 function in colon cancer cells will provide important insight into current therapies targeting VEGFR-1.

Taken together, the present study provides the first evidence that VEGFR-1 plays, at least in part, an important role in cell proliferation through regulating EGF-R.

## 4. Materials and Methods

### 4.1. Reagents and Antibodies

Cycloheximide, MG132, bafilomycin A and DMSO were from Wako Pure chemical (Osaka, Japan). A VEGFR-1 antagonistic peptide (anti-Flt1 peptide; GNQWFI) was from Alpha Diagnostic Int (San Antonio, TX, USA). A VEGFR-2 specific small molecule kinase inhibitor (ZM323881) [19] was obtained from Selleckchem (Houston, TX, USA). Recombinant human EGF was obtained from HIGETA SHOYU (Tokyo, Japan). Recombinant VEGF-A (293-VE), recombinant PlGF (264-PGB) and a neutralizing antibody against human EGF (MAB236) were purchased from R&D Systems (Minneapolis, MN, USA). A neutralizing antibody against human EGF-R (clone LA1) was obtained from Millipore (Burlington, MA, USA). A neutralizing antibody against human VEGFR-1 (Mab0702) was from Novus Biologicals (Oakville, Canada). For flow cytometric analysis, PE-conjugated antibodies against human VEGFR-1 (clone 49560) and against human VEGFR-2 (clone 89106) were purchased from R&D Systems (Minneapolis, MN, USA). For immunoprecipitation analysis, a rabbit polyclonal anti-human VEGFR-1 antibody (C-17) and a mouse monoclonal anti-human EGF-R antibody (528) were from Santa Cruz Biotechnology (Dallas, TX, USA). A rabbit monoclonal anti-phospho-tyrosine antibody (P-Tyr-100) was from Cell Signaling Technology (Danvers, MA, USA). For Westernblot analysis, a rabbit monoclonal anti-human EGF-R antibody (D38B1), a rabbit monoclonal anti-human phospho-EGF-R antibody (D7A5) and a rabbit monoclonal anti-human VEGFR-2 antibody (D5B1) were from Cell Signaling Technology (Danvers, MA, USA). A rabbit monoclonal anti-human VEGFR-1 antibody (Y103) was purchased from Abcam (Cambridge, UK). A rabbit polyclonal anti-phospho-VEGFR-1 (Tyr1213) antibody was purchased from Merck (Darmstadt, Germany). A mouse monoclonal anti-human β-actin antibody (clone AC-74) was from Sigma Aldrich (St. Louis, MO, USA). For immunofluorescence cell staining analysis, an anti-EGF-R antibody conjugated with VioBright FITC was from Miltenyi Biotech (Bergisch Gladbach, Germany).

### 4.2. Cell Culture and Treatment

Human colon cancer cell line (HCT116) were obtained from the American Type Culture Collection (Manassas, VA, USA) and maintained in RPMI1640 medium with 10% fetal bovine serum and antibiotics. For VEGFR-1 or EGF-R activation, 60% confluent cells were treated with control BSA (10 ng/mL), VEGF-A (2 ng/mL), PlGF (10 ng/mL) or EGF (10 ng/mL) for 0.5–48 h before RNA or protein lysates were harvested for further analyses. For VEGFR-1 inhibition, 70%–80% confluent cells were treated with the VEGFR-1 inhibitors; an anti-human VEGFR-1 antibody (2.5 μg/mL) and a VEGFR-1 antagonistic peptide (75 μM). For EGF-R inhibition, 70%–80% confluent cells were treated with the EGF-R inhibitors; an anti-EGF antibody (0.5 μg/mL) and an anti-EGF-R antibody (2.5 μg/mL), or non-immune control IgG (2.5 μg/mL) for 0.5–48 h before RNA or protein lysates were harvested for further analyses.

### 4.3. Cell Proliferation Assay

For proliferation analysis, after serum starvation for 18 h, 50% confluent cells were incubated with the respective inhibitors or ligands for 24–48 h in 0.1% fetal bovine serum (FCS) containing medium. Then, cells were pulsed with 10 μM EdU for 2 h, then fixed with 4% paraformaldehyde. The EdU incorporation was detected using the Click-iT Plus EdU Alexa Fluor 488 Assay Kit, according to the manufacture’s protocol (Thermo Fisher Scientific, Waltham, MA, USA). EdU+ cells from eight to ten randomly chosen fields of at least three independent samples were counted.

### 4.4. Cycloheximide Chase Assay

Cells were pre-treated with control (BSA) or VEGFR-1 ligands (VEGF-A and PlGF) for 1 h, then they were treated with EGF and cycloheximide (25  μg/mL) for the several times. Whole cell lysates were collected and analyzed Western blotting.

### 4.5. Western Blot Analysis

Western immunoblotting was performed as described previously [18]. Total cell lysates were prepared using a lysis buffer containing 100 mM Tris-HCl (pH 6.8), 300 mM NaCl, 2 mM EDTA and 4% (*v*/*v*) SDS. Protein concentrations were determined with Protein assay BCA kit (Nakarai tesque, Kyoto, Japan). Western immunoblotting was performed using a rabbit monoclonal anti-human EGF-R antibody at a 1/5000 dilution, a rabbit monoclonal anti-human phospho-EGF-R antibody at a 1/1000 dilution, a rabbit monoclonal anti-human VEGFR-1 antibody at a 1/1000 dilution, a rabbit polyclonal anti-phospho-VEGFR-1 (Tyr1213) antibody at a 1/5000 dilution, a mouse monoclonal anti-human β-actin antibody at a 1/10,000 dilution. The membranes were developed using ECL Western blot detection reagents (GE Healthcare Life Sciences, Little Chalfont, UK). All immunoblots were performed in triplicate. Immunoblots were imaged using a ChemiDoc imaging system (Bio-Rad Laboratories, Hercules, CA, USA). Quantification was performed with Image J software (NIH, Bethsda, MD, USA). The median pixel intensity quantified for each band was normalized to the loading control. The experimental intensity values of experimental were divided with loading control.

### 4.6. Immunofluorescence Cell Staining

Cells were grown on a glass chamber slide and either was pre-treated with BSA, VEGFR-1 ligands (VEGF-A and PlGF) or VEGFR-1 blockers (anti-VEGFR-1 antibody and VEGFR-1 antagonist) for 1–4 h. Then, cells were blocking with an FcR Blocking Reagent (Miltenyi Biotec, Bergisch Gladbach, Germany) for 15 min at 4 degrees, and an anti-EGF-R antibody conjugated with VioBright FITC was added in the medium for 30 min at 4 degrees. Then, cells were fixed by the addition of 4% paraforlmaldehyde to the culture medium for 15 min. Nuclei were counter stained with 4′,6-diamidino-2-phenylindole (DAPI). Fluorescence images were obtained using a BZ-X710 fluorescence microscope (Keyence Corporation, Osaka, Japan). Magnifications used were ×40 for fluorescence microscopy.

### 4.7. siRNA and Transfection

The siRNAs targeting human EGF-R Mrna (5′-CCAUAAAUGCUACGAAUAU-3′) and human VEGFR-1 mRNA (5′-CAAUCAUAGAUGUCCAAAU-3′) were obtained from Thermo Fisher Scientific (Waltham, MA, USA). Stelth RNAi negative control siRNA (medium GC content, Invitrogen) was used as a control siRNA, which has no homology to human gene products. RNA knockdown analysis was performed as described previously [18]. In brief, cells were transfected with siRNA using Lipofectamine RNAiMax reagent for 24 h (Thermo Fisher Scientific, Waltham, MA, USA), according to the manufacturer’s instructions. Then cell lysates were prepared for immunoblot analysis.

### 4.8. Immunoprecipitation Analysis

Immunoprecipitation analysis was performed as described previously [18]. In brief, cells were harvested in ice-cold lysis buffer (50 mM HEPES, pH 7.0, 150 mM NaCl, 10 mM EDTA, 1.5 mM MgCl_2_, 1% Nonidet P-40, 1% Triton X-100, 1 mM phenylmethylsulfonyl fluoride, 1 mM sodium orthovanadate, 0.5% sodium deoxycholate, 5 mg/mL aprotinin, 5 mg/mL leupeptin, 20 mM sodium fluoride and 20 mM sodium pyrophosphate), homogenized through a 27-gauge needle ten times each, then centrifuged at 16,000× *rpm* for 20 min at 4 degrees. Cell lysates were incubated with a rabbit monoclonal anti-phospho-tyrosine antibody (P-Tyr-100; Cell Signaling Technology) overnight at 4 degrees with constant gentle rocking for immunoprecipitation of tyrosine phosphorylated proteins, followed by the addition of protein G magnet Dynabeads (Thermo Fisher Scientific, Waltham, MA, USA) for 2 h at 4 degrees.

For VEGFR-1/EGF-R co-immunoprecipitation analysis, cells were crosslinked with dithiobis(succinimidyl propionate (DSP) (Thermo Fisher Scientific, Waltham, MA, USA) before cell lysis. Five hundred μg of each clarified lysate was incubated with a rabbit polyclonal anti-human VEGFR-1 antibody (C-17; Santa Cruz Biotechnology) or a mouse monoclonal anti-human EGF-R antibody (sc-120; Santa Cruz Biotechnology) overnight at 4 degrees with constant gentle rocking, followed by the addition of protein G magnet Dynabeads (Thermo Fisher Scientific, Waltham, MA, USA) for 2 h at 4 degrees. Immunoprecipitates were washed, eluted with SDS-PAGE sample buffer, and subjected to 4%–20% polyacrylamide gradient gels for SDS-PAGE. Immunoblot analysis was performed as described above. To exclude the detection of IgG for immunoprecipitation, the TidyBlot HRP conjugated Western blot detection reagent (1:200, Bio-Rad) was used instead of the secondary antibody.

### 4.9. Quantitative RT-PCR

Quantitative RT-PCR was performed as described previously [18]. The levels of transcript for human EGF-R and human GAPDH were measured by quantitative RT-PCR using the following specific primer sets: EGF-R, 5′-TTCCTCCCAGTGCCTGAA-3′(forward) and 5′-GGGTTCAGAGGCTGATTGTG-3′(reverse); GAPDH, 5′-GCTAGGGACGGCCTGAAG-3′ (forward) and 5′-GCCCAATACGACCAAATCC-3′ (reverse). Amplification and quantification of the PCR products were performed using the Applied Biosystems 7500 System (Applied Biosystems; Thermo Fisher Scientific, Waltham, MA, USA). Standards were run in the same plate and the relative standard curve method was used to calculate the relative mRNA expression. RNA amounts were normalized against the GAPDH mRNA levels.

### 4.10. Flow Cytometric Analysis

Cells were harvested and blocked with an FcR Blocking Reagent (Miltenyi Biotec, Bergisch Gladbach, Germany) for 15 min at 4 degrees. Then, cells were stained with a PE-conjugated antibody against VEGFR-1 (clone 49560) or VEGFR-2 (clone 89106) for 30 min at room temperature. For negative control staining, cells were stained with a PE-conjugated isotype control IgG (R&D Systems, Minneapolis, USA). After cells were washed with Flow Cytometry Staining Buffer, flow cytometric analysis was performed using BD FACSVerse flow cytometer (BD Biosciences, Franklin Lakes, San Jose, CA, USA).

### 4.11. Statistical Analysis

Results are expressed as means ± S.D. Statistical analyses of data were done using ANOVA and the Scheffé’s test. *p* value < 0.01 was considered significant.

## Supplementary Materials

The following are available online at https://www.mdpi.com/1422-0067/20/22/5608/s1.

## Abbreviations

VEGF	vascular endothelial growth factor
PlGF	placental growth factor
VEGFR-1	vascular endothelial growth factor receptor-1
EGF	epidermal growth factor
EGF-R	epidermal growth factor receptor
