# Supplementary material for: VEGFR-1 Regulates EGF-R to Promote Proliferation in Colon Cancer Cells
Source: Int J Mol Sci. 2019 Nov 9;20(22):5608. doi: 10.3390/ijms20225608 (PMC6888064; doi:10.3390/ijms20225608)
Supplement: Supplementary file 1 [file ijms-20-05608-s001.pdf]

Supplementary Figure S1. VEGFR-2 is not involved in VEGF-stimulated proliferation.

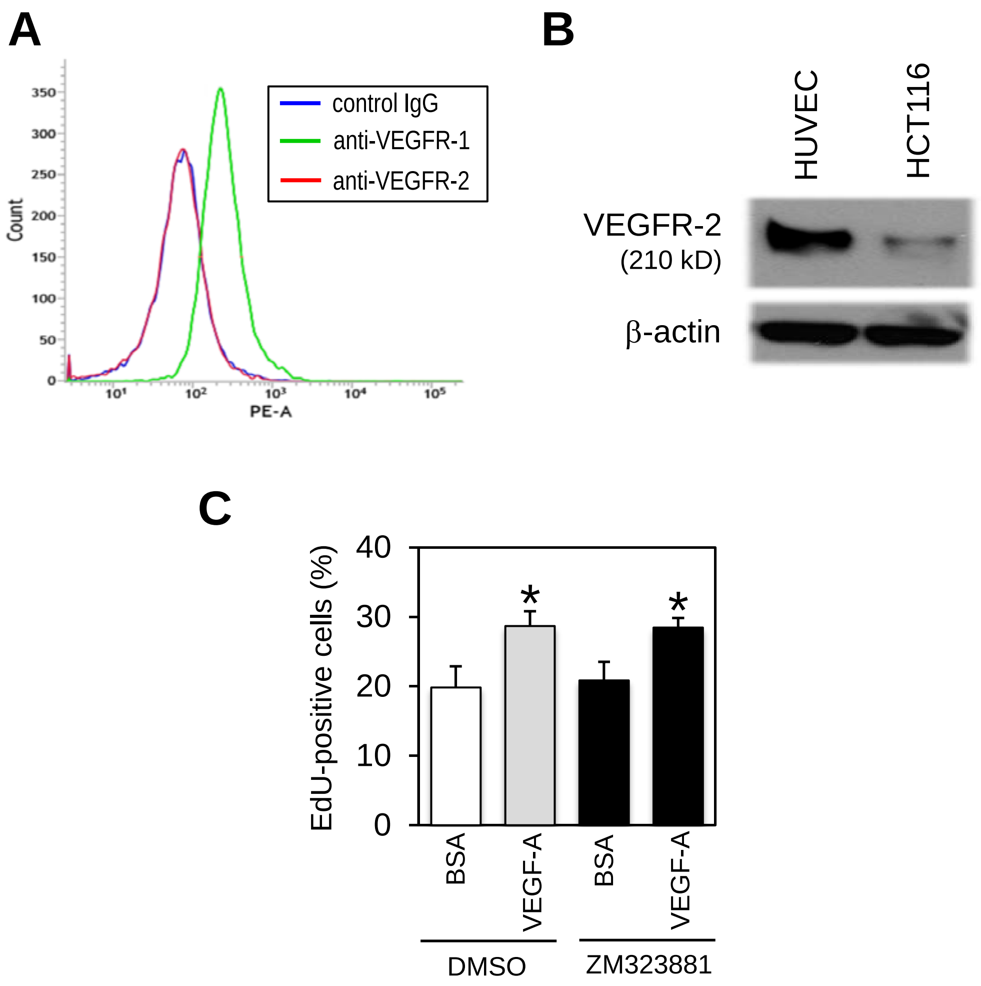

Supplementary Figure S1. VEGFR-2 blockade does not affect the VEGF-A-stimulated proliferation. (A) Cell surface expression of VEGFR-1 (green) and VEGFR-2 (red) was analyzed by FACS analysis. Negative control was analyzed using an irrelevant isotype-matched IgG (blue). (B) VEGFR-2 protein levels in human umbilical vein endothelial cells (HUVEC) and in HCT116 cells were determined by immunoblot analysis. The levels of β-actin are shown as a loading control. (C) Quantification of EdU positive cells under VEGFR-2 inhibiting conditions. Cells were pretreated with a VEGFR-2 specific inhibitor (ZM323881) or DMSO for 1 h, and then treated with BSA or VEGF-A for 24 h. Data are indicated by means ± SD (n=6-8). *P < 0.01, statistically significant increase compared with the BSA-treated control cells.
